# The Future of Disability Research in Australia: Protocol for a Multiphase Research Agenda–Setting Study

**DOI:** 10.2196/31126

**Published:** 2022-01-03

**Authors:** Jennifer Smith-Merry, Mary-Ann O'Donovan, Angela Dew, Bronwyn Hemsley, Christine Imms, Gemma Carey, Simon Darcy, Kathy Ellem, Gisselle Gallego, John Gilroy, Adam Guastella, Manjula Marella, Keith McVilly, Jenny Plumb

**Affiliations:** 1 Centre for Disability Research and Policy Faculty of Medicine and Health The University of Sydney Camperdown Australia; 2 Centre for Disability Studies Faculty of Medicine and Health The University of Sydney Camperdown Australia; 3 Disability and Inclusion, School of Health and Social Development Faculty of Health Deakin University Melbourne Australia; 4 Disability Research Network University of Technology Sydney Sydney Australia; 5 Faculty of Education and Arts The University of Newcastle Newcastle Australia; 6 Murdoch Children's Research Institute The University of Melbourne Melbourne Australia; 7 Centre for Social Innovation University of New South Wales Sydney Australia; 8 School of Nursing, Midwifery and Social Work The University of Queensland Brisbane Australia; 9 Brain and Mind Centre Faculty of Medicine and Health The University of Sydney Sydney Australia; 10 Children's Hospital Westmead Clinical School Faculty of Medicine and Health The University of Sydney Sydney Australia; 11 Nossal Institute for Global Health Melbourne School of Population and Global Health The University of Melbourne Melbourne Australia; 12 School of Social and Political Sciences The University of Melbourne Melbourne Australia

**Keywords:** disability studies, disabled persons, disability research, consumer-driven community-based research, research priorities, mixed methods, research design

## Abstract

**Background:**

For people with disabilities to live a good life, it is essential that funded research in health and social care addresses their interests, meets their needs, and fills gaps in our understanding of the impact that services, systems, and policies may have on them. Decisions about research funding should be based on an understanding of the research priorities of people with disabilities, their supporters and allies, disability researchers, service providers, and policy makers working in the field.

**Objective:**

The aim of this protocol is to describe the research design and methods of a large-scale, disability research agenda–setting exercise conducted in 2021 in Australia.

**Methods:**

The research agenda–setting exercise involves 3 integrated phases of work. In the first phase, a previous audit of disability research in Australia is updated to understand previous research and continuing gaps in the research. Building on this, the second phase involves consultation with stakeholders—people with disabilities and their supporters and family members, the disability workforce, and people working within services and connected sectors (eg, aging, employment, education, and housing), academia, and public policy. Data for the second phase will be gathered as follows: a national web-based survey; a consultation process undertaken through the government and nongovernment sector; and targeted consultation with Aboriginal and Torres Strait Islander people, children with disabilities and their families, people with cognitive disability, and people with complex communication needs. The third phase involves a web-based survey to develop a research agenda based on the outcomes of all phases.

**Results:**

We have started working on 2 parts of the research prioritization exercise. Through the research-mapping exercise we identified 1241 journal articles and book chapters (referred to as *research papers*) and 225 publicly available reports (referred to as *research reports*) produced over the 2018-2020 period. Data collection for the national survey has also been completed. We received 973 fully completed responses to the survey. Analysis of these data is currently underway.

**Conclusions:**

This multi-method research agenda–setting study will be the first to provide an indication of the areas of health and social research that people across the Australian disability community consider should be prioritized in disability research funding decisions. Project results from all phases will be made publicly available through reports, open-access journal publications, and Easy Read documents.

**International Registered Report Identifier (IRRID):**

DERR1-10.2196/31126

## Introduction

### Background

Internationally, there is an increasing need for targeted disability research to align with the changing nature of disability practices, technologies, and policies [1]. In Australia, and internationally, research is needed to inform the implementation of new disability policies and changes to disability systems and services [2]. This is particularly salient now, as new disability-related policies, including the National Disability Insurance Scheme and the revised 10-year National Disability Strategy [3], are being implemented at federal, state, and territory levels. These new strategies and actions mean that disability services and the people they serve must rapidly and continually adapt to new funding, systems, and service structures [4]. Disability research should (1) create new knowledge; (2) encompass the situations of people with disability to address their needs and the issues that are of importance to them; (3) monitor the implementation of new policies; and (4) examine, inform, and affect policy change [5,6]. Furthermore, with finite funding available to support a growing number of active disability researchers in this sector, research resources need to be allocated and used strategically and effectively, by considering both current and emerging issues. Existing prioritization exercises undertaken internationally have been limited in scope and method or conducted only in relation to specific groups or without the participation of people with disability, or other interest groups, and therefore do not represent a broad range of voices [7-9].

**Figure 1:**
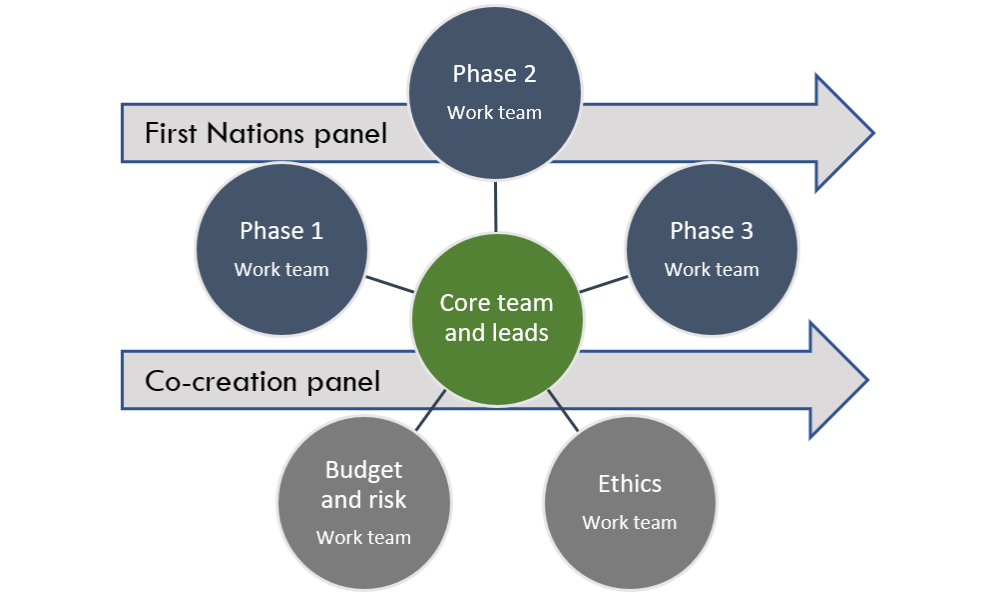
Organization of project phases, advisory panels, and teams.

In 2020, the project team successfully tendered to undertake the research agenda–setting study in a 2-stage expression of interest process judged by a selection panel within the National Disability Research Partnership, which included senior disability researchers, people with disability, supporters, and allies [10]. The project team is a consortium of 31 individuals comprising people with lived experience of disability, family members or other supporters, Aboriginal and Torres Strait Islander people, academic disability researchers, and representatives from nongovernmental organizations (NGOs). A full list of organizations named as part of the tender is shown in Table 1. The team is led by a core project group and administered by the University of Sydney. Members of the consortium work in teams to lead and implement different project phases (Figure 1). Working groups of interested partners were formed from broader consortium membership to develop and advise on each project phase.

Disability, and disability research, is a broad field encompassing a range of sectoral interests, different *diagnostic* or *impairment* groups (eg, spinal cord injury, cerebral palsy, intellectual disability, or autism), or situations (eg, housing, education, employment, health, justice, and citizenship). In addition, disability research has many intersections and overlapping boundaries (eg, in Australia, Aboriginal and Torres Strait Islander people; gender, ethnicity, and lesbian, gay, bisexual, transgender, queer, and intersex [LGBTQI+] communities). Similarly, disability policy is expansive and overlaps with areas of concern for the wider community, such as health, housing, education, employment, leisure, and technology. As such, any attempt at disability research–mapping and agenda-setting must be as broad and inclusive as possible. Audits of Disability Research (2014 and updated in 2017) have mapped the Australian disability research field over the 2000-2017 period [11,12]. These audit reports have been an important resource used by national and international researchers as well as the Australian government and NGOs as a resource for identifying what research exists and can be used in service and policy development [2,13,14]. However, there have been no previous system-wide attempts to set a disability research agenda in Australia.

**Table 1:** The complete list of organizations.

Consortium organizations	Organization category
University of Sydney—Centre for Disability Research and Policy and Centre for Disability Studies (project leads)	Academic research
Ability first	Nongovernment organization
Australian Association of Special Education	Broad-based association
Australian Federation of Disability Organisations	Peak body
Australian National University Lived Research Unit	Academic research
Autism Awareness Australia	Nongovernment organization
Centre for Social Impact National (including University of New South Wales, Swinburne University, University of Western Australia)	Academic research
Children and young people research group (including Murdoch Children’s Research Institute, Monash University, Australian Catholic University)	Academic research
Community Resource Unit	Nongovernment organization
Council of Regional Disability Organisations	Peak body
Deaf Victoria Inc (and Expression Australia)	Nongovernment organization
Deakin University	Academic research
Disability Advocacy Network Australia	Peak body
Disability Research Network, The University of Technology Sydney	Academic research
Family advocacy	Nongovernment organization
Inclusion Australia	Nongovernment organization
Inclusion Melbourne	Nongovernment organization
Kindship	Nongovernment organization
Nossal Institute for Global Health, The University of Melbourne	Academic research
Mobility and Accessibility for Children in Australia Inc.	Nongovernment organization
MND Australia	Nongovernment organization
National Disability Services	Nongovernment organization
Neurodevelopment Australia	Nongovernment organization
Ninti One	Indigenous-owned research nonprofit
NSW Council for Intellectual Disability	Nongovernment organization
Onemda Research and Innovation Centre	Nongovernment organization
Queenslanders with Disability Network	Nongovernment organization
Settlement Services International	Nongovernment organization
University of Melbourne	Academia
University of Queensland	Academia
Vision Australia	Nongovernment organization
Women with Disabilities Australia	Nongovernment organization
Academic advisers: Elizabeth McEntyre, Priscilla Ferazzi, Gerard Goggin	Academic research

### Objectives

In this context, the aim of this research agenda–setting exercise is to map Australian disability research to date, specifically focusing on progress since previous audits, and to identify gaps in the research and areas for further inquiry, to inform decisions regarding the design and funding of disability research programs in Australia [2]. The method and findings may inform the disability sector internationally as other countries move to identify agendas based on the priorities of people with disabilities, their families, and supporters. This research is novel and important as there have been no comparable consultation processes that span all states and territories across one nation, focusing on disability across the life course and encompassing the range of disability or impairment types, sectors, and disability-related issues [8].

## Methods

### Design

**Figure 2:**
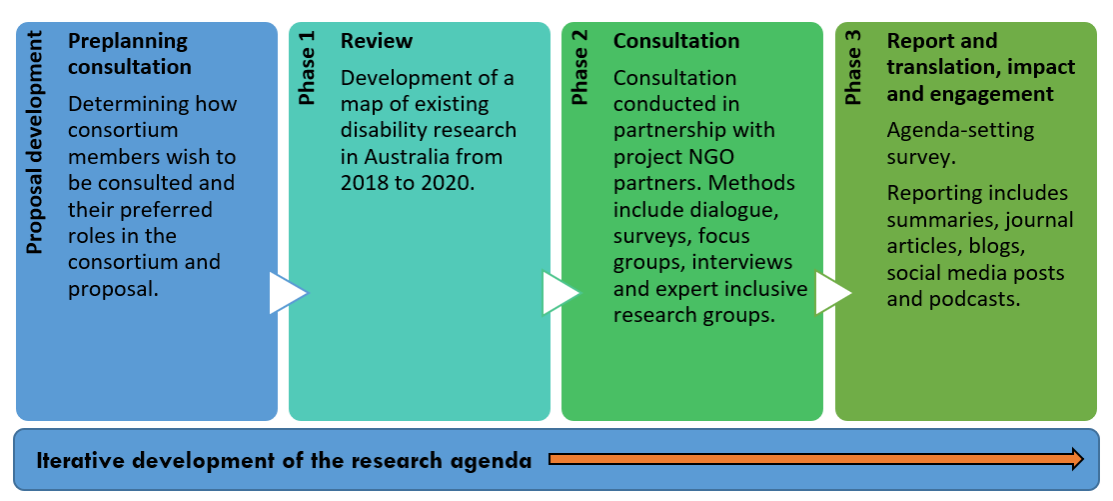
Project design showing the 3 phases of the project. NGO: nongovernmental organization.

This multi-stage study involves research-mapping, community consultation, and agenda-setting exercises (Figure 2). Development of the research agenda will occur iteratively throughout the project, in consultation with the consortium partners, with multiple points of translation of research findings to stakeholders in accessible formats as the disability research agenda develops. In addition to consultation with the project consortium members, the project management structure includes a user-centered cocreation panel with people with disabilities and a First Nations–focused advisory panel, each comprising members of the consortium and others within the broader disability community. These panels are active throughout the project, meeting as needed to provide a focused critical voice to project design and analysis decisions, including development of the project outputs and the final research agenda.

### Preplanning and Project Design Phase

The preconsultation phase involved the consortium collectively and iteratively developing the proposal through a series of meetings. An initial consortium was developed from interested individuals and organizations who then invited other potential consortium members to participate, seeking their advice on ways to design a broader consultation to be attentive and responsive to the needs of people with disabilities and the disability community (completed in September 2020).

### Phase 1: Mapping Existing Research

#### Overview

The research-mapping was designed to (1) determine existing published research and gaps in current research and (2) identify emerging research priorities based on these gaps (data collection completed in May 2021). The consortium members who were leading this phase (Figure 1) applied diverse knowledge and interdisciplinary understanding to question the group’s situated knowledge of disability. These multiple perspectives were important for the relevance of the review approach and to be inclusive of all forms of knowledge to ensure that all views, including nondominant and traditionally excluded views, were heard.

A specified aim of the research agenda was to update the previous audits of disability research with research conducted between 2018 and 2020. The original audits used a conceptual framework based on 8 *domains of everyday life* for people with disability: (1) community and civic participation; (2) economic participation; (3) education; (4) health and well-being; (5) housing and the built environment; (6) safety and security; (7) social relationships; and (8) transport and communication [3,4]. These domains were used to restrict the search into these 8 categories and then as a structure for narrative analysis. In the current mapping process, we did away with these domains as limiters in the search terms, as we felt it would restrict the breadth of disability research, we were able to include in our reporting.

To locate research in peer-reviewed journal articles and books, we systematically searched multiple scientific databases: AMED, Avery, CINAHL, Compendex, Embase, ERIC, Global Health, MEDLINE, PsycINFO, Scopus, Sociological Abstracts, Web of Science Core Collection, and Informit (which includes the following databases: A+Education, Ausport, Families & Society Collection, Humanities & Social Sciences Collection, Literature & Culture Collection, Indigenous Australia, AGIS, FAMILY, APAIS, AMI, AusSportMed, Health & Society Collection, Health Collection, RURAL, Transport Index, ALISA, BUILD, ENGINE, and ARCH). This approach was developed with the assistance of a university librarian with experience in systematic reviews. An example of the search strategy adapted to multiple databases is presented in Textbox 1.

**An example of the search strategy adapted to multiple databases.**

**An example of the search strategy**

(disab* OR handicap* OR mental* retard* OR development* disabilit* OR intellectual disabilit* OR learning disabilit* OR learning disorder* OR hearing impair* OR vision disorder* OR hearing disorder* OR *special needs*) OR (cognitive* disability* OR communication disorder* OR communication disability* OR neurological disorder* OR *brain injury* OR congenital disorder* OR autis* OR *fragile x* OR genetic disorder*) OR (*Cerebral palsy* OR *Spina bifida* OR neurodivers* OR *down syndrome* OR *Fragile X syndrome* OR F*tal Alcohol OR *prenatal alcohol exposure* OR *Rett Syndrome*) OR (psych* disorder* OR psych* disab* OR blind OR vis* impair* OR *low vision* OR *hearing loss* OR *mute OR deaf* OR *sign language* OR *Auslan* OR special education* OR *hard of hearing* OR *attention deficit* OR Tourette*) AND (austral* OR *new south wales* OR south austral* OR west* austral* OR *northern territory* OR *australian capital territory* OR queensland* OR *Tasmania* OR *Victoria*))

To identify research from public reports (sometimes called gray literature), web-based searches using the Google Australia internet search engine were systematically made. A base search string was used, and Australia* disability research filetype:pdf was adapted to the relevant search terms. We undertook a search for partner organizations and other key websites. Any reports that did not contain original data (eg, *how to* guidelines and policy submissions) were excluded from the mapping.

#### Eligibility Criteria for Study Inclusion

The integrative review identified qualitative, quantitative, and mixed methods studies with no limit on study design, data collection methods used, or study quality using the following criteria.

##### Inclusion Criteria

The following were the inclusion criteria:

- Studies published between 2011 and 2020
- Studies published in English in a peer-reviewed journal or published book chapter or as a publicly available report
- A full paper that documented the results of an investigation and secondary analysis of existing data reporting the aim of the investigation, method, findings, and conclusions and recommendations
- At least one aim of the study should be related to people with disability
- Disability research including Australian participants or topics and reporting results on those participants or topics. This included international comparative studies. *Topics* included to capture studies, such as those about Australian disability policy, social context, and services, where there were no participants
- The conclusions derived from results related to people with disability

##### Exclusion Criteria

The following were the exclusion criteria:

- The aims of the paper did not relate to people with disability or disability were mentioned only in passing
- Studies which did not contain original data (eg, commentaries, viewpoints, editorials, or policy documents)
- Studies only discussed disability was acute and transient (eg, rehabilitation from an acute injury, such as short-term limb dysfunction after a fracture)
- Studies included research that was primarily laboratory-based and related to genetics, treatment, diagnosis or cure (eg, medical prevention and cure, surgical or clinical), which did not also consider the broader functioning, disability, health, and well-being of people with disability
- Not a full paper (eg, conference abstracts) or unavailable as full text
- Paper was not written in English

#### Identification and Selection of Studies

The titles and abstracts of all studies generated through the combined database searches were uploaded to the systematic review software Covidence, and duplicates were removed. We used Microsoft Excel to manage research reports. Two team members independently screened all search results against the eligibility criteria in both the abstract and full-text screening phases. Conflicts were resolved by a third reviewer.

The initial list of all relevant papers and reports identified were sent to a large range of disability researchers, NGO partners, and government agencies to identify any papers missed from the search. The list of papers was sent out via the existing networks of consortium members and project advisory groups. Those who received the list were encouraged to send it through their own networks. Any additional papers that were identified were screened according to the method described earlier.

#### Data Analysis

The data extraction task has been shared by multiple members of the research-mapping team based on a standardized data extraction form. The extracted information included the title, year of publication, abstract, study population, focal group of participants, main type of disability being discussed, age group, aim of the paper, topic, primary focus, secondary focus, study design, further details, and study funding sources (Multimedia Appendix 1). These areas were developed collaboratively by the project team to ensure the mapping of key dimensions of disability research in Australia.

The extracted data were then analyzed, integrated, synthesized, and presented both quantitatively (number of papers, domain, age, disability group focus, and study design or type of research) and qualitatively. Narrative synthesis involved identifying (1) main topics within individual studies and synthesizing these across studies, (2) collective limitations of the research scope and methods used (eg, an absence of lived experience-led studies) and knowledge gaps across the studies, and (3) directions for future research across the studies.

### Phase 2: Stakeholder Consultation

In this phase, stakeholders will be consulted using a range of quantitative and qualitative methods to determine their priorities for research, how they use research, and how future research should be shaped so that it is more useful for potential users.

#### Methods of Consultation

The 3 main principles underpinning consultation are *inclusion*, *flexibility*, and *self-determination*. These interlinked principles underpin choices made concerning research methods and their application. It is important to ensure that there are no barriers to participation in the consultation, and this demands flexibility in the approaches used. This includes seeking broad feedback and providing methods and resources that enable people to participate by accommodating their communication and information access needs. Thus, a multi-pronged, multi-stage, multi-platform consultation process has been designed. Nongovernment disabled people’s organizations and advocacy organizations have the best knowledge about their members and, therefore, how to consult with them. Therefore, a consultation toolkit has been designed for use by organizations conducting their own tailored consultations, with resourcing or research support from the consortium as needed. The final approach to consultation by organizations is determined by the communication preferences and styles of their members, using the consultation toolkit.

#### Data Collection

Three main routes of consultation will be used and adapted as needed in different situations.

1. Data collection will be administered by advocacy organizations, disabled people’s organizations, and inclusive research groups. These groups will consult members and stakeholders across Australia, with peak bodies cascading consultations to member organizations. They will use the parts of the consultation toolkit most useful for collecting information from their constituents. Where requested, consortium members can lead consultations on behalf of or with organizations. This is particularly important for small advocacy organizations, who may be restricted in terms of resources to support a consultation.
2. Data collection via a web-based national survey: a national survey has been designed by a subgroup of the consortium, including people with disabilities and advocacy organization partners. It has been designed to be as open as possible in scope and to collect broad perspectives on how research should be designed and conducted and the main topics of interest for the following groups:
  - People with disability, their supporters, and allies
  - Researchers involved in disability research
  - Service providers, disability workforce (eg, disability support workers, health professionals, and educators), policy makers, and others working with people with disabilities (eg, in housing, transport, employment, the arts, health, or education)
  - Anyone who is not otherwise participating in the NGO-focused consultation
  - Data collection for the national survey has been completed.
3. Data collection with First Nations people in regional and metropolitan Aboriginal communities coordinated by the Aboriginal-owned research organization Ninti One. This part of the project is led by JG, who is a leader of the Aboriginal disability scholarship and is a descendant of the Yuin Nation of the New South Wales South Coast. Consultation involves a web-based survey adapted from the national survey. The project has embedded Indigenous Standpoint Theory developed specifically for disability research [15-17].

The consultation has been designed to be adapted to the COVID-19 pandemic restrictions as it can be conducted on the web and face-to-face following safety protocols. Furthermore, the partnership is geographically spread so that local consultations can proceed without the need to travel interstate.

#### Sample and Recruitment

##### Overview

Stakeholders include people with disability and their supporters and family members, the disability workforce, and people working within disability services and connected sectors (eg, aging, employment, education, and housing), academia, and public policy. The proposed sampling strategy is not designed to be representative but rather to reach as many interested people as possible and enable in-depth consultation in relation to their views. The consultation aims to capture the interests of anyone who relates to the concept of disability, rather than using any set definition of inclusion. All partners to the consortium act as a gateway to their respective networks and share the consultation resources throughout these networks by distributing consultation resources and opportunities. Twitter is also used throughout the project to advertise the survey and consultation processes.

##### Developing the Consultation Toolkit

A subgroup of the consortium convened to develop and design the consultation toolkit. On the basis of the discussions across the consortium, data collection templates have been prepared, reviewed, and finalized. The aim was to provide a standardized template for the return of aggregated data to the consortium, while enabling the consultation to be flexible and responsive to the communication and information needs of the participating organizations and their members or stakeholders. Organizations were encouraged to use consultation approaches typically used with their stakeholder groups, with interview and focus group templates provided as guiding documents. Organizations were also responsible for obtaining informed voluntary consent following their usual processes. Templates for participant information sheets and consent forms were developed by the research team, approved by ethics, used in consultations led by consortium and inclusive researchers, and available for organizations if required. Where possible, the resources in the consultation toolkit were adapted from existing codeveloped resources [18,19]. Beyond the working group, draft resources were reviewed internally within 4 organizations (Council for Intellectual disability, Inclusion Melbourne, Deaf Victoria, and People with Disability Australia) in addition to the consortium’s cocreation panel.

##### Elements of the Consultation Toolkit

The final consultation toolkit includes the following resources:

- Easy Read information leaflet
- Guidance on how to complete an interview and a focus group, including preparation and facilitation and example questions that could be asked
- Resource tip sheet for organizations with which to find additional information to support consultations (eg, information on consent and supported decision-making)
- Accessible surveys for different audiences, including video supplementation using Australian Sign Language, to provide context for the consultation and content and purpose of the survey
- A *how* template to be completed and returned by organizations or individuals detailing how the consultation took place, what method was used, and who was included, so the depth and breadth of the consultation could be characterized
- A *what* template to be completed and returned by organizations or individuals collating the findings from the consultation that could inform the agenda-setting task

The information reported in these standardized templates (the *How* and *What* templates) facilitates the process of synthesis by the consortium who go on to bring together the consultation results collected through the different methodologies chosen for individual consultations. The *how* template will act as a quality indicator by reporting the extent to which people with disabilities participate in and facilitate the consultations nationally.

#### Data Analysis Plan

Survey data will be analyzed both quantitative and qualitatively. Quantitative data will be analyzed using Stata for Windows (version 11.0; StataCorp LP). Descriptive statistics will be used to summarize the data. Frequencies and proportions will be calculated. Qualitative data from open-ended text-based responses will be analyzed using a modified thematic analysis that involves an open coding technique [20].

For analysis of the qualitative data, an interpretive approach will be used in analyzing the feedback from each subsection of participants and strands (consultation, survey, and focused data collection). Analysis of the consultation *what* and *how* templates will take a combined deductive and inductive approach of thematic analysis with themes and research priorities contextualized by descriptive data with regard to involvement of people with disabilities and the nature of impairment groupings as recorded in the *how* template [21,22].

The analysis seeks not only to document people’s views but also to develop a deep understanding of the context in which these views have been formulated and the meanings underpinning their perspectives. A report of core findings from each strand will be prepared and shared across the consortium to verify the team’s interpretations. Thematic analysis and triangulation of findings across participant groups will be prepared, and this will form the basis of the phase 3 agenda-setting.

The main output of the consultation phase will be a consultation report that describes in detail the methodology, number of people engaged with through the process, and thematic results of the consultation. Consultation results will be produced in accessible formats, including Australian Sign Language and easy English, as well as ensuring screen reader accessibility. Additional accessibility needs will be met as requested.

### Phase 3: Synthesis of Findings

#### Synthesis and Development of the Research Agenda

This phase will present to the National Disability Research Partnership, a policy- and practice-relevant research agenda. The agenda will bring together the priorities identified from each phase of the project. It will provide commentary on the evidence supporting the inclusion of each identified research priority, its utility for progressing policy and practice, advancing rights and enabling the flourishing of people with disability.

The third phase is informed by the James Lind Alliance Priority Setting Partnership methodology [23,24]. This is a detailed cocreation methodology used internationally since 2004 to set research agendas in an equal partnership between lay people, people affected by health conditions, and support people and professionals.

Phase 3 will involve consolidating the evidence from phases 1 and 2 using thematic consolidation and a comparison of findings to create overarching research themes that have been disproportionately underresearched or not researched at all in the Australian context in the past 10 years; of where publications do exist, but where findings have not been communicated or translated for use at the system, community, or individual level (evidence-practice gap); that are considered priorities for future research by nonresearchers including people with disability.

The themes, which are yet to be identified, will be presented in a web-based survey to stakeholders, including representatives of those groups and individuals who took part in phase 2 consultations and those who indicated their interest in participating in phase 3. Purposive sampling for participants not represented through this opt-in process will be conducted through the consortium, NGOs, governments, and research networks. We aim for 500-1000 responses, which is feasible given the response rate for the phase 2 survey that was completed by almost 1000 respondents. The survey will focus on high-level research themes and ask respondents to rank and comment on the importance and applicability of these themes from the following perspectives:

- Effectiveness of research into policy and practice related to each of the themes
- Enabling factors identified by research related to each of the themes
- Experiences explored in research about everyday life and outcomes for people with disability

#### Data Analysis Plan

Survey responses will be analyzed using the same quantitative and qualitative data analysis processes used for the phase 2 survey to produce the disability research agenda.

The main output of this final phase will be a report on identifying the research agenda themes.

#### Public and Patient Involvement

People with lived experience of disability and their supporters and allies will be involved in all phases of the project, from the beginning of the development of the tender documents and research plan. The research question underpinning this project concerns what should be prioritized in disability research in Australia. This question was developed by the National Disability Research Partnership, which includes people with disabilities. Within this project consortium, the strategies for data collection, analysis, and dissemination have all been developed in partnership with people with disabilities, including core project team members with disabilities, family members, and supporters.

#### Ethics

This project necessitates an enhanced ethical review because of the potential vulnerability of the participants. The coproduction of all aspects of data collection is an important part of the ethical approach. Ethics approval was obtained for the phase 2 survey and other consultation processes from the University of Sydney Human Research Ethics Committee (2021/175, 2021/318, 2021/443). The phase 3 survey is currently under ethical consideration. All participants in the research will be requested to provide written consent to participate in the interviews and focus groups. For surveys, consent will be obtained by acknowledgment of reading the participant information sheet and submission of the survey. Only full survey data (where the participant has fully completed and submitted the survey) will be used. For individuals (eg, children and people with very complex intellectual disability) who do not have the legal ability to fully consent for themselves, their guardians will be requested to provide consent, but we will also ask for consent from the participants themselves.

## Results

The project was launched in October 2020, and data collection has begun in phases 1 and 2. This project is currently in progress. We have completed the research-mapping phase and co-designed the consultation documents for dissemination across participating organizations. The national survey has been completed.

The main project outputs will be (1) full, accessible reports of all stages and (2) summary documents that explain the research agenda in a briefer format. These will be created in several accessible formats. Summary reports will be distributed to the disability community using email and team websites. Social media (eg, Twitter, Facebook, and LinkedIn) will be used to disseminate project outcomes and gather feedback progressively throughout the project, using the hashtag #AusDisAbilityResearch. Specific research articles will report all phases of the project, including the mapping and consultation phases.

## Discussion

Disability research has the potential to radically improve the lives of people with disability if it is targeted to those areas that are priorities for people with disability, service providers, policy makers, and others across the disability sector [10,25]. Currently, there is no way of knowing what the nation’s disability research priorities are, leading to a reliance on what researchers, government agencies, and funding bodies consider important based on their own domains, or areas of expertise or responsibility. This multi-method, research agenda–setting study will provide an indication of what people across the Australian disability community, including people with disability, consider should be prioritized in disability research [26].

Given that research outputs and impacts will ultimately inform structures for research funding priorities (and in turn policy), it is imperative that these outputs and outcomes have validity, confirmability, transferability, and verifiability across the disability community [6,9]. Communication with the disability community and the inclusion of as many voices as possible are essential to the project process. This need has underpinned decisions about the cross-sector consortium and project organization. It has also underpinned the decision to conduct consultations primarily through NGO partners. Key elements of the research findings will be released throughout the project to engage the disability sector, including people with disability, in the project as it develops and encourages participation in the consultation.

## Appendix

Multimedia Appendix 1 Data extraction template from Covidence.

## Abbreviations

LGBTQI+: lesbian, gay, bisexual, transgender, queer, and intersex
NGO: nongovernmental organization

## References

[1] Shakespeare T (2015). Disability Research Today International Perspectives.

[2] Nasir MN, Hussain RB (2019). Disability research production in Malaysia: a call for critical discussion and emancipatory praxis. Int J Stud Child Women Elderly Disabled People.

[3] A new national disability strategy. Australian Government Department of Social Services.

[4] Carey G, Dickinson H, Malbon E, Weier M, Duff G (2020). Burdensome administration and its risks: competing logics in policy implementation. Administration Soc.

[5] Stevenson M (2010). Flexible and responsive research: developing rights-based emancipatory disability research methodology in collaboration with young adults with down syndrome. Australian Soc Work.

[6] Stone E, Priestley M (1996). Parasites, pawns and partners: disability research and the role of non-disabled researchers. Br J Sociol.

[7] Priestley M, Waddington L, Bessozi C (2010). Towards an agenda for disability research in Europe: learning from disabled people’s organisations. Disability Soc.

[8] The national institute on disability, independent living and rehabilitation research 2018-2023 long-range plan. Department of Health and Human Services USA.

[9] Pratt B (2021). Achieving inclusive research priority-setting: what do people with lived experience and the public think is essential?. BMC Med Ethics.

[10] National Disability Research Partnership 2021. NDRP.

[11] Audit of Disability Research in Australia. Centre for Disability Research and Policy.

[12] Audit of disability research in Australia update 2017. Centre for Disability Research and Policy.

[13] Tilbury C, Hughes M, Bigby C, Osmond J (2015). Social work research in the child protection field in Australia. Br J Soc Work.

[14] Wilson NJ, Macdonald J, Hayman B, Bright AM, Frawley P, Gallego G (2018). A narrative review of the literature about people with intellectual disability who identify as lesbian, gay, bisexual, transgender, intersex or questioning. J Intellect Disabil.

[15] Gilroy J, Dew A, Lincoln M, Ryall L, Jensen H, Taylor K, Barton R, McRae K, Flood V (2018). Indigenous persons with disability in remote Australia: research methodology and Indigenous community control. Disability Soc.

[16] Gilroy J, Donelly M (2016). Australian indigenous people with disability: ethics and standpoint theory. Disability in the Global South.

[17] Gilroy J, Donelly M, Colmar S (2016). Twelve factors that can influence the participation of Aboriginal people in disability services. Aust Indigenous Health Bull.

[18] Have Your Say: Consultation Survey Launched for United Nations Convention on the Rights of Persons with Disabilities Shadow Report. Disabled People's Organisations Australia.

[19] Jenkin E, Wilson E, Clarke M, Campain R, Murfitt K (2017). Listening to the voices of children: understanding the human rights priorities of children with disability in Vanuatu and Papua New Guinea. Disability Soc.

[20] Joffe H (2011). Thematic analysis. Qualitative Research Methods in Mental Health and Psychotherapy: A Guide for Students and Practitioners.

[21] Azungah T (2018). Qualitative research: deductive and inductive approaches to data analysis. Qual Res J.

[22] Braun V, Clarke V (2006). Using thematic analysis in psychology. Qual Res Psychol.

[23] Morris C, Simkiss D, Busk M, Morris M, Allard A, Denness J, Janssens A, Stimson A, Coghill J, Robinson K, Fenton M, Cowan K (2015). Setting research priorities to improve the health of children and young people with neurodisability: a British Academy of Childhood Disability-James Lind Alliance Research Priority Setting Partnership. BMJ Open.

[24] The James Lind Alliance. The James Lind Alliance Priority Setting Partnerships.

[25] National Disability Research and Development Agenda. Australian Government.

[26] Woolf SH, Zimmerman E, Haley A, Krist AH (2016). Authentic engagement of patients and communities can transform research, practice, and policy. Health Aff (Millwood).

