# Supplementary material for: The Future of Disability Research in Australia: Protocol for a Multiphase Research Agenda–Setting Study
Source: JMIR Res Protoc. 2022 Jan 3;11(1):e31126. doi: 10.2196/31126 (PMC8764607; doi:10.2196/31126)
Supplement: Multimedia Appendix 1 [file resprot_v11i1e31126_app1.docx]

## Supplementary materials: Data extraction template

**General information**

**Title** Copy and paste title of paper.

**Year of publication**

**Abstract** Copy and paste abstract.

**Study population**

**Aboriginal and Torres Strait Islander people - focused study**

Does the research focus on Aboriginal and Torres Strait Islander people in either the overall research topic or conclusions (i.e. not just mentioned in passing)?

- Yes
- No

**Culturally and linguistically diverse. If Yes state which groups if mentioned**

Leave blank if not a focus.

**Focal group of study**

Who is the group that is the focus of the paper? If there is no specific focus group (e.g. just talking about disability generally, then leave blank).

- People with disability (provide further details below)
- Family and informal carers
- Siblings and children of people with disability
- Workforce and staffing (provide further details below)
- Other

**Main type of disability being discussed**

What is the main type of disability being discussed?

- General discussion of disability
- Intellectual disability
- Developmental disability
- Brain injury
- Mental illness
- Spinal cord injury
- Autism
- ADHD
- Cerebral Palsy
- Other
- Age group
- Please select the most relevant age group for the study.
- Infants and early childhood (<5)
- Children (5 < 18)
- Adolescents
- Children of any age
- Young adults (18 < 30)
- Adults of any age
- Older age (65+)
- People of all ages/ age not specified
- Other

**Topic of study**

**Aim of paper**

Provide a very brief description of the aim of the paper (e.g. aim is to discuss the implications of using the ICF for measuring functioning of people with disability in employment).

**Topic descriptive text**

Provide a very brief description of the overarching topic (e.g. teacher attitudes towards inclusion of students with disability in early education).

**Primary focus topic**

Provide the primary focus of the study (not just topics mentioned in passing). Pick only one primary focus from the list. You can provide a secondary focus topic in the next section. If you need more than two focus topics, please put it in the notes at the bottom of the data extraction template.

- Education (see below to provide more detail) Any type of education – so can include school, home schooling, higher ed, vocational education
- Self-determination, choice and control Study focus is on the extent to which people can make their own decisions or are empowered to make their own decisions.
- Employment, volunteering, work experience Any employment related to people with disability and family/carers. Workforce issues related to disability workers not included here. This is included below.
- Retirement, ageing, aged care, end of life.
- Economic security and social security payments. Includes research related to welfare and social security, poverty.
- Service development and evaluation. Where the focus of the study is on any service providing support to people with disability, family, carers (e.g. health, allied health, disability services etc.)
- Workforce development and leadership Focus is on the workforce providing services to people with disability e.g. training of disability support workers, medical practitioners, workforce shortages etc.
- Disability accommodations (see below) Accommodations provided to people with disability, including interpretation.
- Communication needs
- Policy Includes NDIS, NDS, education policy relating to disability, housing policy related to disability etc.
- Technology Includes apps, wearable devices, ipads, computers
- Transport
- Housing Includes studies focusing on types of accommodation and homelessness.
- Quality of Life
- Lived experiences of disability: Personal experiences of people with disability and family members, supporters, allies and unpaid/informal caregivers.
- Social and community involvement, Leisure, sport and physical activity
- Health and wellbeing Includes the health and wellbeing needs of people with disability and their interactions with health care systems. Includes pharmacology.
- Social care (see below for further details)
- Epidemiology Studies which are primarily focused on prevalence of disability or a particular phenomenon in a population.
- Public attitudes to people with disability Attitudes to disability, stigma, disability confidence.
- Sexuality and reproductive rights
- Behaviour support
- Safety, security, discrimination and abuse
- Law, criminal justice and rights Includes the legal system, discussion of the UN CRPD, human rights, restraint and seclusion etc.
- Media - traditional and social
- The arts, culture and literature
- Critical theory, philosophy and religion
- Intersectional approaches (e.g. race and disability, gender and disability)
- Disability Research and research methods Includes papers that are about disability research and research methods as the primary topic. E.g. review of disability research, analysis of inclusive research practices.
- Other

**Secondary focus topic (**Only use this topic if there is a second main topic).

- Education (see below to provide more detail)
- Self-determination, choice and control
- Employment, volunteering, work experience
- Retirement and ageing
- Economic security and social security payments
- Service development and evaluation
- Workforce development and leadership
- Disability accommodations and communication (see below)
- Policy
- Technology and transport
- Housing
- Quality of life
- Lived experiences of disability
- Social and community involvement, leisure and sport
- Health and wellbeing (see below for further details))
- Social care (see below for further details)
- Epidemiology
- Public attitudes to people with disability
- Sexuality
- Safety, security, discrimination and abuse
- Law, criminal justice and rights
- Media - traditional and social
- The arts, cultural and literature
- Critical theory, philosophy and religion
- Intersectional approaches (e.g. race and disability, gender and disability)
- Other

**Study design**

- Randomised controlled trial
- Systematic review
- Narrative, scoping reviews
- Service evaluation
- Qualitative research
- Prevalence study
- Case report
- Policy analysis
- Co-research, inclusive research
- Participatory action research
- Economic evaluation
- Other

**More specific details**

**Education - further details 1**

Choose the main level of education being discussed.

- Early childhood
- Primary school
- Secondary school
- higher education
- vocational education
- transitions between education levels
- All/ no specific levels
- Other

**Education - further details 2**

Provide further information about the area of education research. Pick main topic. If more than one then put the second topic in the 'other' box.

- Inclusion
- Teacher experiences
- Student experiences
- Parent experiences
- Teacher/ teacher assistant training
- Education policy
- accommodations and technology
- behaviour
- Other

**Health and Social Care - further details.**

What type of health or social care services are being discussed.

- Acute hospital based care
- Community mental health care
- Primary care (including GPs)
- Pharmacy
- Dentistry
- Allied Health
- Rehabilitation
- Supported accommodation
- Long-term care facilities
- Respite
- Supported employment
- Other

**Workforce - further details**

- disability support workers
- nurses
- doctors
- allied health (OT, Physios etc)
- dentists
- educators
- psychologists
- social workers
- Other

**Accommodations - further details**

- augmentative and alternative communication
- braille
- Auslan
- Wheelchairs, mobility aids
- accessible housing
- accessible transport
- work accommodations
- education accommodations
- Wearable and implantable technology (e.g. microswitches)
- Computers and ipad related technology
- Other

**Study funding sources** (as noted in paper)

**Notes, including other important data not captured in the above.**

Please include here anything not captured above.
